# Development and Performance Assessment of Single- and Double-Layer TbAG:Ce and YAG:Ce Composite Scintillators on GAGG:Ce Substrates for Optimized α–γ Discrimination and Pulse-Shape Analysis

**DOI:** 10.3390/ma19102001

**Published:** 2026-05-12

**Authors:** Abdellah Bachiri, Agnieszka Syntfeld-Każuch, Vitalii Gorbenko, Sandra Witkiewicz-Lukaszek, Tetiana Zorenko, Yurii Syrotych, Lukasz Adamowski, Lukasz Swiderski, Vasyl Stasiv, Yaroslav Zhydachevskyy, Yuriy Zorenko

**Affiliations:** 1 National Centre for Nuclear Research, 05-400 Otwock, Poland; agnieszka.syntfeld@ncbj.gov.pl (A.S.-K.); lukasz.adamowski@ncbj.gov.pl (L.A.); lukasz.swiderski@ncbj.gov.pl (L.S.); 2 Department of Physics, Kazimierz Wielki University in Bydgoszcz, 85-090 Bydgoszcz, Poland; gorbenko@ukw.edu.pl (V.G.); s-witkiewicz@wp.pl (S.W.-L.); tzorenko@ukw.edu.pl (T.Z.); syr@ukw.edu.pl (Y.S.); 3 Faculty of Physics, Warsaw University of Technology, Koszykowa 75, 00-662 Warsaw, Poland; 4 Institute of Physics, Polish Academy of Sciences, Aleja Lotników 32/46, 02-668 Warsaw, Poland; stasiv@ifpan.edu.pl (V.S.); zhydach@ifpan.edu.pl (Y.Z.); 5 Medical Physics Department, Prof. Franciszek Łukaszczyk Oncology Center, 85-796 Bydgoszcz, Poland

**Keywords:** composite scintillators, single-crystalline garnet films, GAGG:Ce, pulse-shape analysis, α–γ discrimination, liquid-phase epitaxy

## Abstract

In this work, we report the fabrication and characterization of single-film and double-film composite epitaxial garnet structures based on single-crystalline films (SCFs) and bulk single-crystal (SC) scintillators for enhanced α–γ discrimination in mixed radiation fields. These composite scintillators consist of TbAG:Ce and YAG:Ce SCFs grown by liquid-phase epitaxy (LPE) on Czochralski-grown Gd_3_Ga_2.5_Al_2.5_O_12_ (GAGG:Ce) bulk SC substrates. Single- and double-film architectures were designed to optimize the energy absorption and pulse-shape discrimination (PSD) performance for low-penetrating α-particles and high-energy γ-rays. Energy calibration was performed using different γ-ray sources (^57^Co, ^51^Cr, and ^137^Cs), enabling the conversion of detector signals to a calibrated electron-equivalent energy scale (keVee). Integration gates were systematically optimized, yielding maximum figures of merit (FOM) of 1.4 for the GAGG:Ce SC substrate, 1.9 for the single-film composite, and 5.0 for the double-film composite, demonstrating a progressive improvement in α–γ discrimination with increasing structural complexity. Two-dimensional PSD density maps reveal well-separated α and γ events, with the highest separation observed for the double-film composite. These results indicate that the engineering of LPE-grown composites provides tunable scintillation decay profiles, enhanced temporal separation, and increased light yields, making them promising candidates for applications such as mixed radiation field detection, dosimetry, and radiation monitoring.

## 1. Introduction

The development of advanced luminescent materials based on single-crystalline films (SCFs) grown on bulk single-crystal (SC) substrates has attracted significant attention for radiation detection applications [1,2]. The liquid-phase epitaxy (LPE) method is one of the most effective techniques for the fabrication of such SCFs, as it enables the growth of high-quality films with controlled thicknesses, sharp epitaxial interfaces, and excellent optical properties. As a result, LPE-grown SCFs are widely employed in cathodoluminescent screens [3,4], laser media [5,6], and scintillators for the detection of α- and β-particles and low-energy X- or γ-ray quanta, as well as scintillating screens for microtomography detectors using X-ray sources and synchrotron radiation [7,8].

An important advantage of the LPE method is the possibility of fabricating advanced composite scintillators of the phoswich (“phosphor sandwich”) type, designed for the registration and discrimination of different components of ionizing radiation [9,10,11]. Such structures typically consist of one or more SCFs for the detection of low-penetrating α- or β-particles combined with a bulk SC substrate for highly penetrating X- or γ-rays. Owing to the epitaxial SCF/SC interface and closely matched refractive indices, optical losses are minimized, resulting in improved scintillation signal separation and enhanced particle discrimination. In addition, the SCF thickness can be tailored to the penetration depth of incident radiation. For example, thicknesses of approximately 12–15 µm are sufficient to fully absorb α-particles emitted by common radioisotopes such as ^239^Pu (5.15 MeV) and ^241^Am (5.5 MeV).

The physical basis of α–γ discrimination in such systems arises from differences in energy deposition and scintillation decay kinetics. γ-rays generate low-ionization-density electron tracks, whereas α-particles produce dense ionization over short paths, resulting in different fast-to-slow scintillation component ratios. This principle is exploited in the charge comparison pulse-shape discrimination (PSD) method used in this work, where integration over short- and long-time gates enables the statistical separation of radiation types based on the temporal response.

Previous studies have demonstrated the successful fabrication of LPE-grown composite scintillators based on YAG:Ce SCFs, including single-film composite scintillator and double-film composite scintillator structures, which exhibited efficient α–γ discrimination through differences in scintillation decay kinetics [9]. However, the relatively low density (ρ = 4.5 g·cm^−3^) and effective atomic number (Z_eff_ = 29) of YAG limit their applicability mainly to low-energy radiation detection. Therefore, garnet-based materials with a higher density and higher Z_eff_, such as TbAG and GAGG, are of particular interest. These materials, with ρ = 6.07 and 6.63 g/cm^3^ and Z_eff_ = 34 and 54.5, respectively, are promising candidates for mixed fields of α-particles and high-energy γ-quanta. Furthermore, engineering the SCF composition, thickness, and activator doping (e.g., Ce^3+^, Pr^3+^, Sc^3+^) enables control over scintillation kinetics and energy transfer processes, providing a route toward improved composite scintillators [12,13]. In parallel, alternative scintillator systems such as oxide and halide perovskites have also been explored to tailor the luminescence and trapping properties [14,15,16,17].

Recent advances (2024–2025) have further demonstrated that multilayer epitaxial garnet structures grown by LPE significantly improve α–γ discrimination through engineered scintillation decay kinetics and optimized energy deposition profiles [18,19]. Continued progress in Ce-doped garnets has also deepened our understanding of the relationship between the crystal structure, defect chemistry, and scintillation performance [20]. These developments highlight the growing interest in tailored composite scintillator architectures for enhanced radiation discrimination.

In this work, two types of composite scintillators were fabricated using 500 µm thick Gd_3_Al_2.5_Ga_2.5_O_12_:Ce (GAGG:Ce) single-crystal substrates. The first configuration is a single-film composite scintillator consisting of a 10 µm Tb_3_Al_5_O_12_:Ce (TbAG:Ce) SCF deposited on the substrate. The second configuration is a double-film composite scintillator, in which a 10 µm TbAG:Ce SCF is covered by an additional 20 µm Y_3_Al_5_O_12_:Ce (YAG:Ce) SCF. Both composite scintillator architectures are designed for α–γ discrimination [9]. The double-film composite scintillator is expected to provide an improved figure of merit due to enhanced energy deposition control and the more effective separation of scintillation signals.

## 2. Materials and Methods

### 2.1. Sample Preparation

The liquid-phase epitaxy (LPE) technique used in this work is a well-established and reliable method for the growth of SCF scintillators, widely employed for garnet-based materials. The process ensures high reproducibility due to precise control of key growth parameters such as the temperature, supersaturation, the melt composition, and the growth rate. In our experiments, these parameters were carefully controlled and kept constant across all growth runs. The investigated TbAG:Ce and YAG:Ce SCFs, as well as the composite structures, were prepared in multiple independent growth processes under identical conditions.

**Figure 1:**
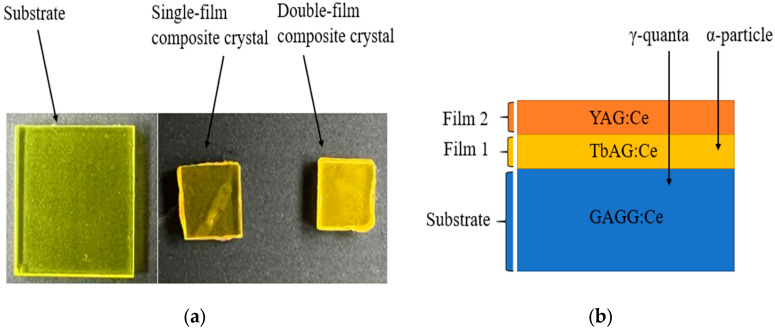
(**a**) GAGG:Ce single-crystal (SC) substrate scintillator and composite scintillators with one and two epitaxially grown single-crystalline films (SCFs). (**b**) Schematic illustration of the layered composite scintillator architecture.

The composite scintillators investigated in this work were based on TbAG:Ce SCFs and YAG:Ce SCFs grown by the LPE method onto GAGG:Ce bulk SC substrates. A schematic illustration of the investigated structures is shown in Figure 1. The commercially available GAGG:Ce SC substrates were grown by the Czochralski method at approximately 1850 °C in an Ar + 1% O_2_ atmosphere. Two types of composite scintillator architectures were fabricated:

- A single-film composite scintillator consisting of a 10 µm thick TbAG:Ce SCF grown on a GAGG:Ce SC substrate;
- A double-film composite scintillator comprising a GAGG:Ce SC substrate and a 10 µm thick TbAG:Ce SCF covered with a 20 µm thick YAG:Ce SCF.

The SCF thicknesses were selected to ensure the efficient absorption of low-penetrating α-particles, while the GAGG:Ce SC substrate serves as an efficient detector for high-energy γ-rays. The single-film composite configuration increases the interaction volume and enables the temporal separation of scintillation signals originating from the individual layers, providing a suitable platform for investigating the influence of SCF stacking on scintillation behavior and α–γ discrimination.

The Ce^3+^ concentration in the investigated materials was approximately 0.098 at.% for TbAG:Ce, 0.11 at.% for YAG:Ce, and 0.018 at.% for GAGG:Ce. The light yield (LY) under ^137^Cs γ-ray excitation was 41,900 photons/MeV for GAGG:Ce [21], while it was not measured for the SCFs due to their limited thicknesses. However, the LYs of the SCF and SC substrates were measured under α-particle excitation from a ^239^Pu source in comparison with a standard YAG:Ce sample with an LY of 2650 photons/MeV.

A summary of the key physical and compositional parameters of the composite scintillators is provided in Table 1.

### 2.2. Reproducibility and Scalability of the LPE Growth Process

**Figure 2:**
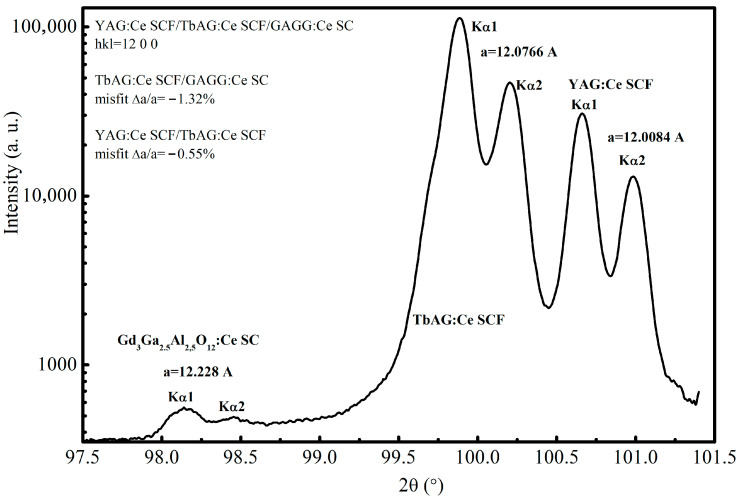
XRD pattern of the (12 0 0) plane of the YAG:Ce SCF/TbAG:Ce SCF/GAGG:Ce substrate epitaxial structure grown on a GAGG substrate with (100) orientation. The corresponding lattice misfits, defined as $m=(a_{f}-a_{s})/a_{s}\times 100\%$, are −0.55% for the YAG:Ce SCF/TbAG:Ce SCF interface and −1.32% for the TbAG:Ce SCF/GAGG:Ce interface.

The obtained samples exhibited consistent structural quality, as confirmed by the X-ray diffraction (XRD) pattern of the selected YAG:Ce SCF/TbAG:Ce SCF/GAGG:Ce SC epitaxial structure (Figure 2), comparable surface morphology, and reproducible optical and scintillation properties. In particular, the measured light yield and decay kinetics were consistent within experimental uncertainty, typically within ±5–10% across independently prepared samples in one LPE growth circle.

Regarding scalability, the LPE method is inherently suitable for the growth of large-area films and multilayer structures, as demonstrated in previous studies on garnet scintillators and optical materials. The thickness of the SCFs can be reproducibly controlled in the micrometer range by adjusting the growth time, making this technique compatible with the scalable fabrication of composite scintillators.

Although a full statistical analysis of sample-to-sample variation was beyond the scope of the present work, the consistency of the results obtained for independently prepared samples confirms the good reproducibility and reliability of the applied growth process.

### 2.3. Methods

The scintillation properties of the investigated samples—namely, a bulk GAGG:Ce SC substrate, a single-film composite scintillator (a GAGG:Ce SC substrate covered with a TbAG:Ce SCF), and a double-film composite scintillator (a GAGG:Ce substrate covered step by step, firstly with a TbAG:Ce SCF and later with a YAG:Ce SCF)—were systematically studied under α- and γ-irradiation at room temperature (RT). The measurements included the relative light yield, radioluminescence (RL) spectra, time-resolved scintillation pulse shapes, and pulse-shape discrimination (PSD) for particle identification. All samples were optically coupled to a photodetector and measured in light-tight enclosures to suppress background illumination. This methodology enabled the direct comparison of the scintillation performance, scintillation decay characteristics, and particle discrimination capabilities among the different scintillator configurations. Detailed descriptions of the individual measurement setups are provided in the following subsections.

#### 2.3.1. α- and γ-Ray-Induced Light Emission and Radioluminescence Spectrometry

The absorption spectra and the cathodoluminescence (CL) and X-ray-excited radioluminescence (RL) spectra, as well as the scintillation light yield (LY) and scintillation decay kinetics, were recorded at room temperature (RT) to characterize the optical, luminescent, and scintillation properties of the GAGG:Ce SC substrate and single-film composite and double-film composite scintillators. The absorption spectra of the crystals were measured using a Jasco V730 spectrophotometer. A JEOL JSM-820 scanning electron microscope (SEM) equipped with a StellarNet grating spectrometer operating in the 200–1120 nm spectral range was used to record the CL spectra. RL spectra were measured using the registration channel of a Horiba Jobin-Yvon Fluorolog-3 spectrofluorometer equipped with a Hamamatsu R928P PMT in photon-counting mode. The RL spectra were corrected for the spectral response of the detection system. Samples were irradiated using a 130 kV microfocus L9181-02 X-ray source positioned 50 mm from the Be window.

In the first step, the scintillation LY and decay kinetics of the GAGG:Ce SC substrate and the TbAG:Ce SCF/GAGG:Ce SC (single-film composite) and YAG:Ce SCF/TbAG:Ce SCF/GAGG:Ce SC (double-film composite) structures were measured using a setup involving a Hamamatsu H6521 photomultiplier module (PMT), a multichannel analyzer, and a digital TDS3052 oscilloscope with a shaping time of 12 μs under excitation with α-particles from a ^239^Pu (5.15 MeV) source. The LYs of the SCF and crystals under study were compared with that of a reference YAG:Ce SCF with a light yield of 2650 photons/MeV. All these measurements were performed at RT.

Later, the scintillation response was investigated under α-particle and γ-ray excitation at RT. A ^241^Am source provided α-particles, while 662 keV photons from a ^137^Cs source were used for γ-ray measurements. Signals were detected with a Hamamatsu R6231-100 photomultiplier tube (PMT) operated at 1250 V. Samples were optically coupled to the PMT window with Viscasil optical grease. For γ-ray measurements, the samples were wrapped in Teflon tape to maximize the light collection efficiency, whereas, for α-particle excitation, the samples were left unwrapped to avoid the attenuation of α-particles. The PMT anode signal was processed using a Canberra 2005 charge-sensitive integrating preamplifier and amplified with a Canberra 2022 spectroscopy amplifier (shaping time 2 µs). Owing to the high quantum efficiency of the PMT photocathode near the Ce^3+^ emission wavelength (~550 nm), scintillation pulses were efficiently detected. Pulse-height spectra were recorded with a TUKAN-8K-USB multichannel analyzer (MCA) [22], and the relative LY was determined by comparison to the GAGG:Ce SC substrate as a reference.

#### 2.3.2. Light Pulse-Shape Measurements

**Figure 3:**
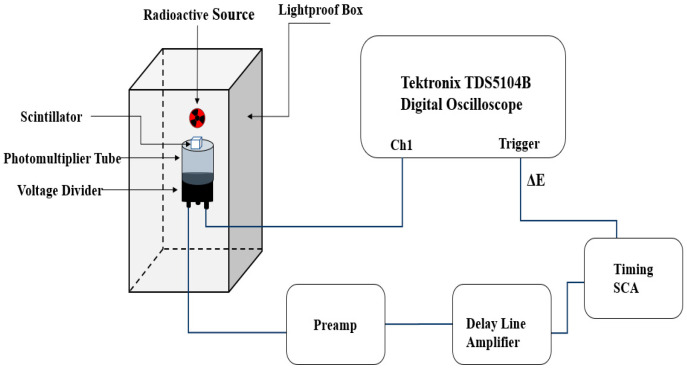
Schematic diagram of the experimental setup used for measurement of scintillation light pulse decay at a defined energy window.

Scintillation time profiles were measured under α-particle and γ-ray excitation to investigate the temporal characteristics of the emitted light. A fast Hamamatsu R5320 PMT (transit time 10 ns, rise time 0.7 ns, time jitter 140 ps) detected emitted photons. The anode signal was recorded with a Tektronix TDS5104B digital oscilloscope (1 GHz bandwidth, 5 GS/s sampling rate), while the last dynode signal defined an energy window (ΔE) and triggered data acquisition. The dynode output was amplified using a Canberra 2005 charge-sensitive preamplifier and an ORTEC 460 delay line amplifier (DLA). One output of the DLA was connected to the TUKAN-8K-USB MCA to record pulse-height spectra within the energy window, and the bipolar output was sent to an ORTEC 551 timing single channel analyzer (TSCA). The TSCA output triggered the oscilloscope, and a secondary output was connected to an ORTEC 416A gate and delay generator to synchronize MCA acquisition. Energy windows were set to capture events at the 662 keV γ-ray full-energy peak from ^137^Cs and the α-particle peak near 5.5 MeV from ^241^Am. A schematic of the setup is shown in Figure 3.

#### 2.3.3. Pulse-Shape Discrimination

**Figure 4:**
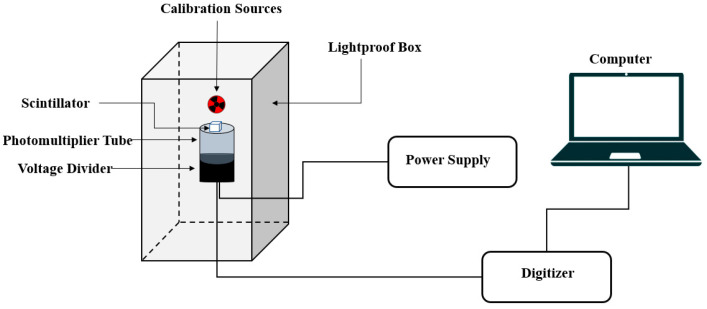
Layout of the experimental setup used for α–γ discrimination measurements.

PSD measurements distinguished α-particle and γ-ray interactions based on differences in scintillation decay kinetics. PMT signals were digitized using a CAEN DT5730 waveform digitizer (14-bit resolution, 500 MS/s sampling rate, 2 V dynamic range) with digital pulse processing (DPP) firmware for multiparametric acquisition. This allowed the simultaneous recording of the pulse height and pulse shape. The samples included the GAGG:Ce SC substrate and composite scintillators based on the single-film composite TbAG:Ce SCF/GAGG:Ce SC epitaxial structure and double-film composite YAG:Ce SCF/TbAG:Ce SCF/GAGG:Ce SC epitaxial structure. All samples were optically coupled to the PMT as described above and mounted vertically in a light-tight enclosure, 3 mm from the α-particle or γ-ray source. PSD was performed using ^241^Am and ^137^Cs sources. The experimental setup is shown in Figure 4.

PSD parameters were extracted using the charge comparison method (CCM). In this approach, two integrals of the PMT current pulse are calculated: a long integration gate ($Q_{long}$), encompassing the entire scintillation pulse, and a short integration gate ($Q_{short}$), covering only the initial part of the pulse. The PSD parameter is defined as

(1) $$PSD=1-\frac{Q_{short}}{Q_{long}}$$

**Figure 5:**
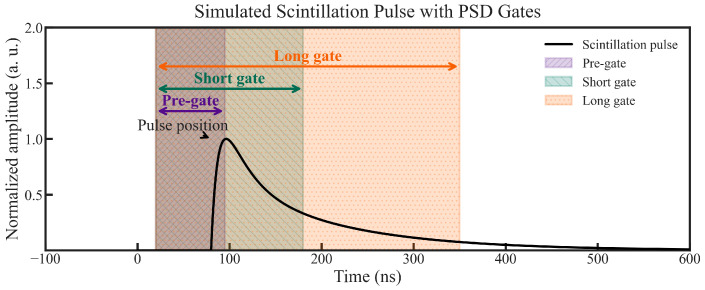
Diagram of the α/γ discrimination method implemented in the CAEN DT5730 digitizer. The pre-gate duration of 50 ns contributes to the total length of each integration gate.

Differences in scintillation decay kinetics between α-particle and γ-ray interactions result in distinct PSD values, enabling effective particle discrimination. The implementation of the CCM using the CAEN DT5730 digitizer is illustrated in Figure 5 [23].

## 3. Results and Discussion

### 3.1. Absorption Spectra

**Figure 6:**
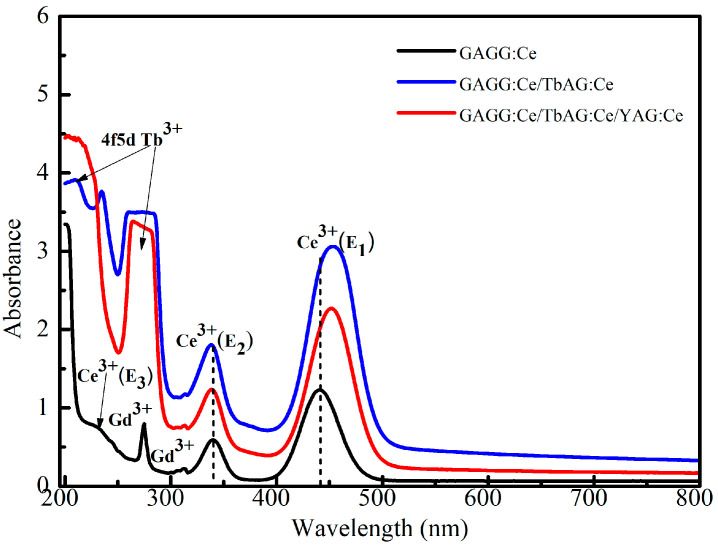
RT absorption spectra of the GAGG:Ce SC substrate (black curve) and the TbAG:Ce SCF/GAGG:Ce SC (single-film composite, red curve) and YAG:Ce SCF/TbAG:Ce SCF/GAGG:Ce SC (double-film composite, blue curve) composite scintillators.

The room-temperature (RT) absorption spectra of the GAGG:Ce substrate (black curve) and two composite scintillators based on the TbAG:Ce SCF/GAGG:Ce SC (single-film composite, red curve) and YAG:Ce SCF/TbAG:Ce SCF/GAGG:Ce SC (double-film composite, blue curve) epitaxial structures are presented in Figure 6.

All spectra exhibit characteristic absorption features originating from the Ce^3+^ activator, as well as from Gd^3+^ and Tb^3+^ host cations. Specifically, the sharp absorption bands located near 275 nm and 313 nm (low intensity) are attributed to the intra-configurational ^8^S_7_/_2_ → ^6^I_J_ and ^8^S_7_/_2_ → ^6^P_J_ transitions of Gd^3+^ ions in the GAGG host [24]. The broad bands peaking around 210 nm and 260–270 nm correspond to the 4f → 4f5d transitions of Tb^3+^ ions in the TbAG host.

The broad absorption bands observed in the near-UV and blue spectral regions are assigned to the allowed 4f–5d transitions of Ce^3+^ ions. In particular, the band centered at approximately 338–340 nm corresponds to the E_2_ transition, while the band located in the 440–455 nm region is associated with the lower-energy E_1_ transition. An additional high-energy Ce^3+^ absorption peak detected at 230–235 nm (E_3_) is attributed to the 4f–5d (T_2_g) transitions [25,26,27].

In comparison with the bulk GAGG:Ce substrate, the single- and double-film composite scintillators reveal additional broad absorption bands centered at approximately 210 nm and 264–280 nm. The intensity of these bands increases with the number of deposited SCF layers, indicating their origin within the epitaxial films. These bands are assigned to the 4f → 4f5d transitions of Tb^3+^ ions in the TbAG host.

Due to the spectral overlap between the Gd^3+^ absorption band at ~275 nm and the Tb^3+^ 4f–5d band around 264–280 nm, the Gd^3+^-related feature becomes progressively less pronounced in the composite samples, particularly in the double-film composite scintillator. The relative enhancement of the Ce-related absorption bands in the composite configurations further reflects the contribution of the SCF layers to the overall optical responses of the composites [25].

### 3.2. CL and RL Emission Spectra

**Figure 7:**
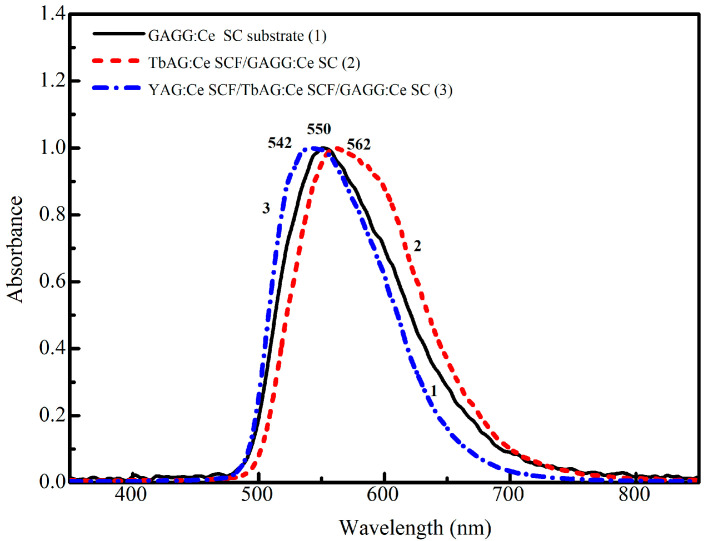
CL spectra of Gd_3_Al_2.5_Ga_2.5_O_12_:Ce SC substrate (1), TbAG:Ce SCF/GAGG:Ce SC (single-film composite, 2), and YAG:Ce SCF/TbAG:Ce SCF/GAGG:Ce SC (double-film composite, 3) scintillators.

The normalized cathodoluminescence (CL) spectra of the GAGG:Ce SC substrate, TbAG:Ce SCF, and YAG:Ce in the respective single-film composite and double-film composite scintillators are shown in Figure 7. The dominant doublet luminescence bands peaking at 550–560 nm in the spectra of all SCFs and substrates correspond to the 5d_1_ → 4f (^2^F_5_/_2_; ^2^F_7_/_2_) transitions of Ce^3+^ ions in the YAG, TbAG, and GAGG garnet hosts. The different positions of the maxima of the Ce^3+^ emission bands at 542, 550, and 562 nm in the CL spectra of the YAG:Ce SCF, TbAG:Ce SCF, and GAGG:Ce substrate, respectively, are caused by the different crystal field strengths and Stokes shifts, which are equal to 0.407, 0.53, and 0.474 eV, respectively, in the corresponding garnet hosts.

The normalized X-ray-excited radioluminescence (RL) spectra of the GAGG:Ce SC substrate (1), TbAG:Ce SCF/GAGG:Ce SC (single-film composite, 2), and YAG:Ce SCF/TbAG:Ce SCF/GAGG:Ce SC (double-film composite, 3) scintillators were recorded over the wavelength range of 300–800 nm (Figure 7). All samples exhibit dominant luminescence bands associated with the Ce^3+^ 5d_1_ → 4f (^2^F_5_/_2_, ^2^F_7_/_2_) transitions in the garnet host lattice.

**Figure 8:**
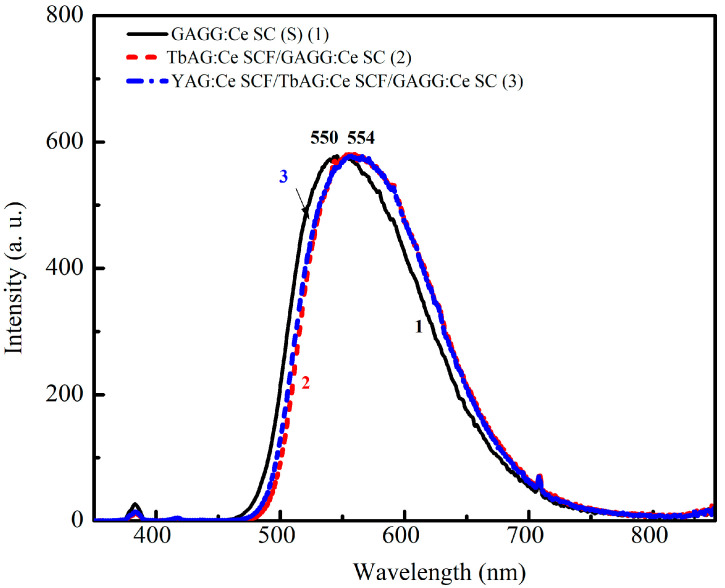
RL spectra of the GAGG:Ce SC (1) substrate and TbAG:Ce SCF/GAGG:Ce SC (single-film composite, 2) and YAG:Ce SCF/TbAG:Ce SCF/GAGG:Ce SC (double-film composite, 3) scintillators.

The bulk GAGG:Ce SC substrate shows a broad emission band peaking at approximately 550 nm. The presence of the TbAG:Ce SCF results in a noticeable red shift in the Ce^3+^-related emission band to about 554 nm, in agreement with the corresponding CL spectra (Figure 7). Additional coverage of the single-film composite structure with a YAG:Ce SCF leads to a slight (by a few nm) blue shift in the RL spectrum of the double-film composite due to the contribution of the YAG:Ce SCF, consistent with Figure 7. Meanwhile, the RL emission spectra of the single- and double-film composites are generally similar (Figure 8), indicating that the luminescence response under highly penetrating excitation is dominated by the GAGG:Ce substrate.

### 3.3. Relative Light Yields and Decay Times of SCF and Substrate Scintillators

The scintillation properties of the GAGG:Ce SC substrate, as well as the TbAG:Ce SCF/GAGG:Ce SC and YAG:Ce SCF/TbAG:Ce SCF/GAGG:Ce SC composite scintillators, were investigated under α-particle excitation from a ^241^Am (5.5 MeV) source and γ-ray excitation from a ^137^Cs source (662 keV). The relative LY values were derived from PHS and are summarized in Table 2, together with the scintillation decay parameters obtained under α- and γ-ray excitation. Notably, the composite scintillators exhibit significantly different decay times from those of the substrate. This feature is exploited at the signal analysis stage to achieve the enhanced pulse-shape discrimination (PSD) of α- and γ-ray-induced events.

For the correct interpretation of the results, it should be noted that all light yield (LY) values reported in this work are relative and independently normalized within each excitation type to the response of the GAGG:Ce SC substrate measured under identical electronic conditions. All measurements were performed using the same acquisition settings (amplifier gain, shaping time, and digitizer configuration), ensuring full internal consistency across samples. However, α- and γ-excitation measurements were carried out under different experimental geometries: α-particle measurements were performed in a direct irradiation geometry without optical wrapping to avoid additional energy losses and scattering effects, while γ-ray measurements were performed with Teflon wrapping to maximize the light collection efficiency for penetrating radiation. Therefore, LY values should be compared only within the same excitation type (α or γ) and not directly between α- and γ-induced responses.

#### 3.3.1. Relative Light Yield

**Figure 9:**
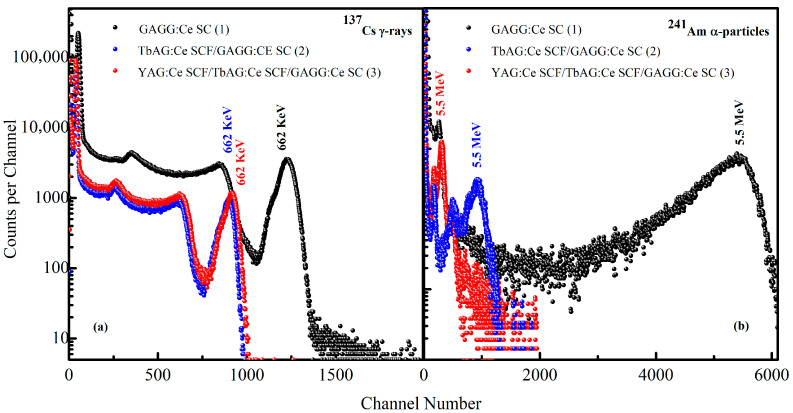
Pulse-height spectra of the GAGG:Ce SC substrate and the TbAG:Ce SCF/GAGG:Ce SC (single-film composite) and YAG:Ce SCF/TbAG:Ce SCF/GAGG:Ce SC (double-film composite) scintillators under γ-ray excitation from a ^137^Cs source (**a**) and α-particle excitation from a ^241^Am source (**b**).

The PHS registered for the GAGG:Ce SC substrate and the TbAG:Ce SCF/GAGG:Ce SC (single-film composite) and YAG:Ce SCF/TbAG:Ce SCF/GAGG:Ce SC (double-film composite) scintillators under excitation by α-particles from a ^241^Am source and γ-rays from a ^137^Cs source are shown in Figure 9. All measurements were performed under identical experimental conditions using the same amplifier gain (0.5 × 5) and a shaping time constant of 2 µs.

Under α-particle excitation, the main PHS peaks correspond to the total absorption of 5.5 MeV α-particles (Figure 9a). The bulk GAGG:Ce SC substrate exhibits the highest scintillation LY, with the full-energy α peak located at the 5440 channel (LY = 100%). For the single-film composite, the α-related peak shifts to the 930 channel, with a reduced LY of 17%, indicating that a substantial fraction of the α-particle energy is absorbed in the TbAG:Ce SCF with a thickness of 10 μm. In the double-film composite, the α peak is further shifted to the 315 channel, with a significantly lower LY of 5.8%, demonstrating that nearly all α-particles are absorbed within the upper YAG:Ce SCF with a thickness of 20 μm. The reduced LY of the SCF-containing samples is attributed to the lower scintillation efficiency of the LPE-grown films prepared from PbO-based fluxes. Specifically, the crystallization of these films from such melt solutions is associated with Pb^2+^ incorporation and the formation of Pb^2+^–Ce^4+^ charge-compensated centers, leading to the partial quenching of Ce^3+^-related luminescence [27]. The influence of the substrate and film thickness on the relative LY is further supported by previous observations.

In contrast, under γ-ray excitation at 662 keV (Figure 9b), the main PHS peaks correspond to the total absorption of γ-quanta. The γ full-energy peak of the GAGG:Ce SC substrate is observed at the 1225 channel (LY = 100%). For the single-film and double-film composite scintillators, the corresponding peaks are located at the 913 and 898 channels, corresponding to relative LYs of 75% and 73%, respectively. The relatively small shift in the γ-related peaks compared to the substrate indicates that γ-rays predominantly excite the GAGG:Ce substrate due to their high penetration depth. Consequently, the scintillation response under γ-ray excitation is mainly governed by the Ce^3+^-doped GAGG substrate, while the influence of the SCF layers on the total light yield remains limited.

The combined effects of Ce^3+^ activator distribution, Pb^2+^-related quenching in the SCFs, and the composite scintillator geometry result in a strong contrast between the LY responses under γ- and α-particle excitation. This contrast is most pronounced for the double-film structure, confirming the suitability of the investigated composite scintillators for efficient α–γ discrimination.

#### 3.3.2. Scintillation Decay Time

**Figure 10:**
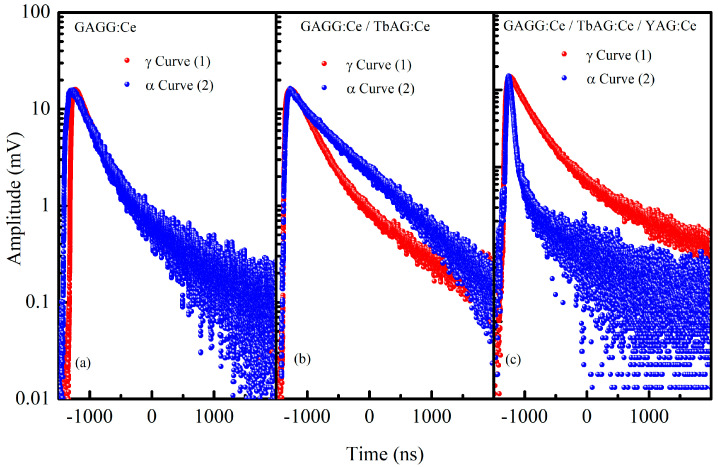
Scintillation decay profiles of the investigated samples under α- and γ-excitation. Panel (**a**)—GAGG:Ce SC substrate; panel (**b**)—TbAG:Ce SCF/GAGG:Ce SC (single-film composite); panel (**c**)—YAG:Ce SCF/TbAG:Ce SCF/GAGG:Ce SC (double-film composite). Curves 1 correspond to α-particle excitation from a ^241^Am source, and curves 2 correspond to γ-ray excitation from a ^137^Cs source.

The scintillation decay curves of the GAGG:Ce SC substrate and the TbAG:Ce SCF/GAGG:Ce SC (single-film composite) and YAG:Ce SCF/TbAG:Ce SCF/GAGG:Ce SC (double-film composite) scintillators were measured under excitation by α-particles from a ^241^Am source and γ-rays from a ^137^Cs source. The extracted decay times are summarized in Table 2, and the corresponding scintillation decay curves under γ- and α-particle excitation are shown in Figure 10. The experimental decay profiles were analyzed using a multi-exponential fitting function of the form

(2) $$y\left(x\right)=y_{0}+\sum_{i=1}^{n}A_{i}exp\left[-\frac{x-x_{0}}{\tau_{i}}\right]$$

where $A_{i}$ and $\tau_{i}$ denote the amplitudes and decay time constants of the individual scintillation components, respectively, and $y_{0}$ represents the background contribution. In the present study, the scintillation decay curves were satisfactorily described by a two-exponential model (n = 2) for all investigated samples. To enable a quantitative comparison of the scintillation kinetics, the mean decay time $\tau_{mean}\;$ was calculated from the fitted parameters in accordance with Equation (3), proposed by Zatryb and Klak [28], as given by

(3) $$\tau_{mean}=\frac{\sum_{i=1}^{n}A_{i}{\tau_{i}}^{2}}{\sum_{i=1}^{n}A_{i}\tau_{i}}$$

The relative contribution of each decay component was determined from its integrated intensity. Since the time integral of an exponential term is proportional to $A_{i}\tau_{i}$, the percentage contribution of the i-th component was calculated as

(4) $$\frac{A_{i}\tau_{i}}{\sum_{i=1}^{n}A_{i}\tau_{i}}\times 100\%$$

The decay curves of the three samples illustrate how the SCF configurations influence the scintillation kinetics. For the GAGG:Ce SC substrate, the γ- and α-decays have similar shapes, with a fast component around 253–303 ns and a slower component near 1030–1180 ns, resulting in largely overlapping overall decay profiles.

The curves show a gradual decline dominated by the slower tail, reflecting the substrate’s intrinsic scintillation behavior (τ_γ_mean_ = 459 ns, τ_α_mean_ = 456 ns). With a single TbAG:Ce SCF (single-film composite), the decay shapes change noticeably. The γ-decay retains a relatively fast initial drop (309 ns) followed by a moderate tail (770 ns), while the α-decay is dominated by the slow component (752 ns), giving the overall curve a longer tail (τ_α_mean_ = 708 ns). This indicates that the SCF modifies the energy relaxation pathways, enhancing the contribution of the slow scintillation component for α-excitation.

For the double TbAG:Ce SCF + YAG:Ce SCF structure (double-film composite), the decay curves exhibit even more pronounced differences in shape. The γ-decay shows a moderate fast component (255 ns) and a prominent slow component (825 ns), whereas the α-decay has a sharp initial drop (50 ns) followed by a moderate tail (582 ns), producing a faster initial response compared to γ (τ_α_mean_ = 205 ns). The evolution of the decay shapes from the substrate to the single-film composite to the double-film composite demonstrates that SCF stacking significantly modifies the relative contributions of fast and slow scintillation components, particularly under α-excitation.

This pronounced fast α-component in the double-film structure originates from the preferential energy deposition of α-particles in the upper YAG:Ce film, where the higher excitation density and more efficient carrier capture by fast Ce^3+^ 5d–4f radiative transitions dominate the early-time scintillation response. In addition, the multilayer architecture introduces enhanced interface-related carrier migration and trapping–detrapping processes, which further redistribute the excitation energy toward faster decay channels compared to γ-ray excitation, occurring mainly in the GAGG:Ce substrate.

These observations indicate that the scintillation decay shapes provide clear information about the dynamics of energy deposition and relaxation. Based on these differences, pulse-shape discrimination measurements can be performed to distinguish α- and γ-radiation, and the results are expected to demonstrate progressively improved discrimination capabilities with increasing complexity in the composite scintillator structure.

### 3.4. Pulse-Shape Discrimination Performance

Pulse-shape discrimination (PSD) was evaluated for the GAGG:Ce SC substrate and the TbAG:Ce SCF/GAGG:Ce SC (single-film composite) and YAG:Ce SCF/TbAG:Ce SCF/GAGG:Ce SC (double-film composite) scintillators. First, energy calibration was performed using γ-ray sources (^57^Co, ^51^Cr, and ^137^Cs), while α-particles were excluded due to strong quenching effects. Using the calibrated signals, integration gates were optimized to extract PSD parameters and calculate the figure of merit (FOM) for α–γ separation for each sample.

#### 3.4.1. Energy Calibration

The measured photopeak positions for the GAGG:Ce SC substrate were 94.99 channels (^57^Co, 122 keV), 251.9 channels (^51^Cr, 320 keV), and 532.37 channels (^137^Cs, 662 keV). A linear fit yielded the calibration equation

E (keVee) = 1.2328 × Channel + 6.6747(5)

demonstrating excellent linearity across the investigated energy range. The same procedure was applied to the composite samples to determine their sample-specific calibration parameters. These calibration relations were subsequently used to convert all pulse-height spectra and two-dimensional PSD maps into calibrated energy units.

The resulting calibration equations are as follows.

- Single-film composite scintillator:

E (keVee) = 1.0620 × Channel + 20.6959 (6)

- Double-film composite scintillator:

E (keVee) = 1.4013 × Channel − 5.5932 (7)

**Figure 11:**
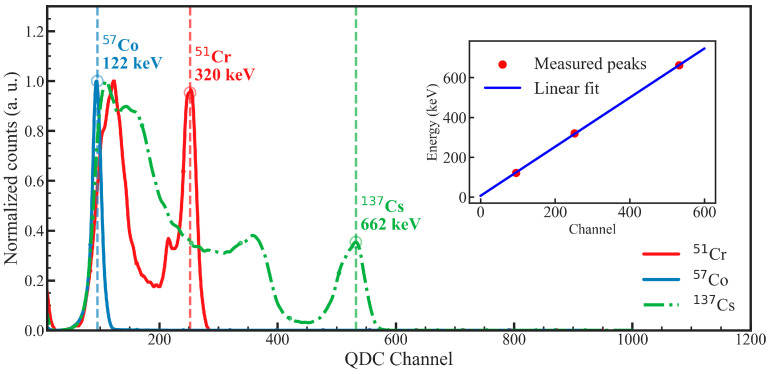
Energy calibration of GAGG:Ce SC substrate using ^57^Co, ^51^Cr, and ^137^Cs γ-ray peaks.

The full spectra, identified photopeaks, and corresponding linear calibration fit for the GAGG:Ce SC substrate are shown in Figure 11 as a representative example.

#### 3.4.2. PSD Evaluation of Substrate and Composites

The PSD performance strongly depends on the choice of integration gate used to evaluate the scintillation pulse shape. A fixed pre-gate of 50 ns was applied to all measurements to account for baseline subtraction.

Energy windows for α–γ discrimination were defined individually for each sample based on the calibrated energy scale (Section 3.4.1) and analysis of the two-dimensional PSD versus energy distributions (Figures 14–16), by selecting regions where both α- and γ-induced events are clearly visible and well separated, while maintaining sufficient counting statistics:

- 592–956 keVee for the GAGG:Ce substrate;
- 70–358 keVee for the single-film composite;
- 22–218 keVee for the double-film composite.

**Figure 12:**
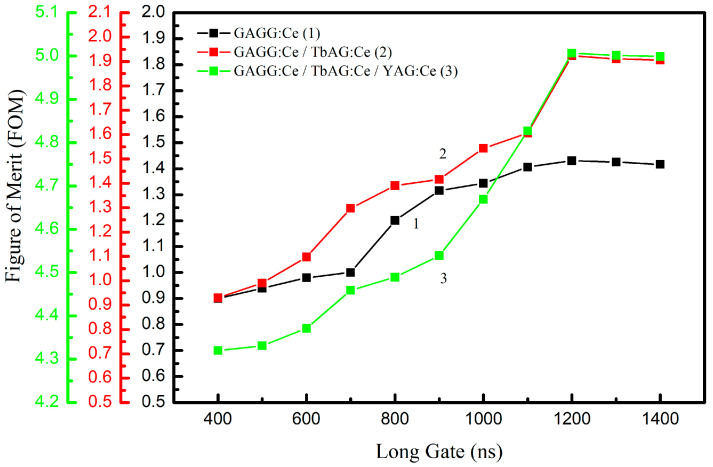
FOM as a function of the long-gate width (400–1400 ns) for the GAGG:Ce SC substrate, TbAG:Ce SCF/GAGG:Ce SC single-film composite scintillator, and YAG:Ce SCF/TbAG:Ce SCF/GAGG:Ce SC double-film composite scintillator. Optimal long-gate values of 1200 ns were identified for all samples.

Within these calibrated energy ranges, the figure of merit (FOM) for α–γ separation was calculated as

(8) $$FOM=\frac{peak\;separation}{{FWHM}_{\gamma }+{FWHM}_{\alpha }}$$

where FWHM represents the full width at half maximum of the peaks corresponding to α-particle and γ-ray detection, projected onto the PSD parameter axis. This FOM provides a quantitative measure of the detector’s discrimination capabilities. The optimization of the integration gates was carried out in two steps. In the first step, the long integration gate was varied from 400 ns to 1400 ns in 100 ns increments, while the short gate was kept constant. The FOM for α–γ separation, calculated using Equation (8), was extracted from the resulting PSD distributions for each gate width (Figure 12). Optimal long-gate values of 1200 ns were identified for all samples.

**Figure 13:**
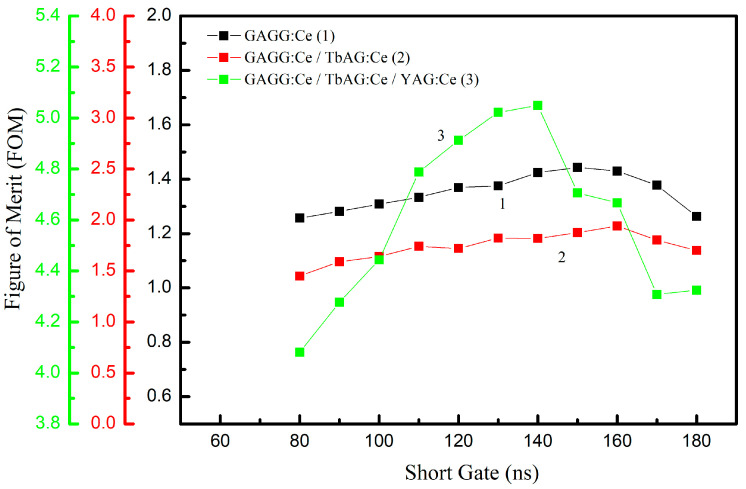
FOM as a function of the short-gate width (80–180 ns) at the optimal long-gate value for each sample. Maximum FOM values: 1.4 (GAGG:Ce SC substrate), 1.9 (TbAG:Ce SCF/GAGG:Ce SC single-film composite scintillator), 5.0 (YAG:Ce SCF/TbAG:Ce SCF/GAGG:Ce SC double-film composite scintillator).

In the second step, the short integration gate was varied from 80 ns to 180 ns in 10 ns increments, while the long gate was fixed at its optimal value for each sample. The resulting FOM values (Figure 13) show that the short gate has the strongest influence on the discrimination performance. The maximum FOM values increase progressively with the addition of scintillating SCF components:

- 1.4 for the GAGG:Ce SC substrate;
- 1.9 for the TbAG:Ce SCF/GAGG:Ce SC single-film composite scintillator;
- 5.0 for the YAG:Ce SCF/TbAG:Ce SCF/GAGG:Ce SC double-film composite scintillator.

This trend demonstrates that adding TbAG:Ce and YAG:Ce scintillating crystal films enhances α–γ discrimination by improving the separation between the respective populations in the PSD space.

**Figure 14:**
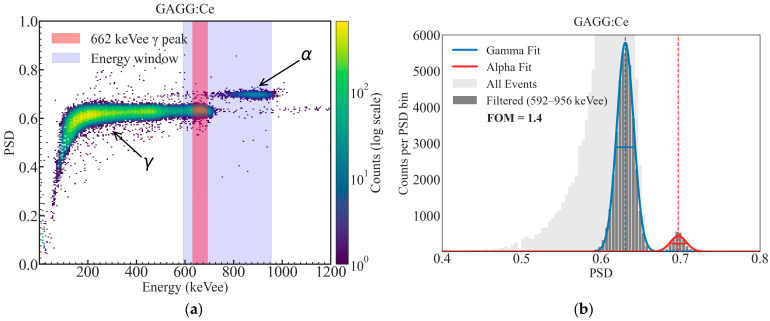
Two-dimensional PSD versus energy histogram (**a**) and corresponding one-dimensional PSD projection (**b**) with Gaussian fits and figure-of-merit (FOM) evaluation for GAGG:Ce SC substrate measured using ^241^Am and ^137^Cs sources.

**Figure 15:**
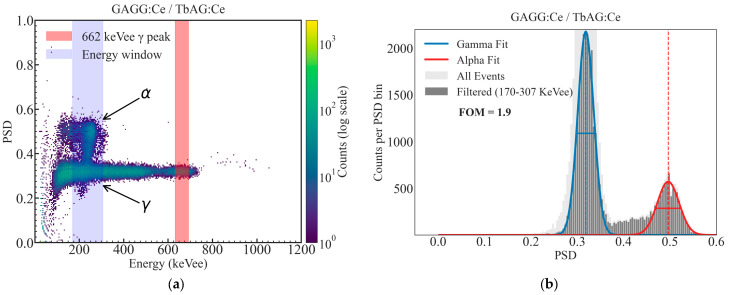
Two-dimensional PSD versus energy histogram (**a**) and corresponding one-dimensional PSD projection (**b**) with Gaussian fits and figure-of-merit (FOM) evaluation for TbAG:Ce SCF/GAGG:Ce SC single-film composite scintillator measured using ^241^Am and ^137^Cs sources.

**Figure 16:**
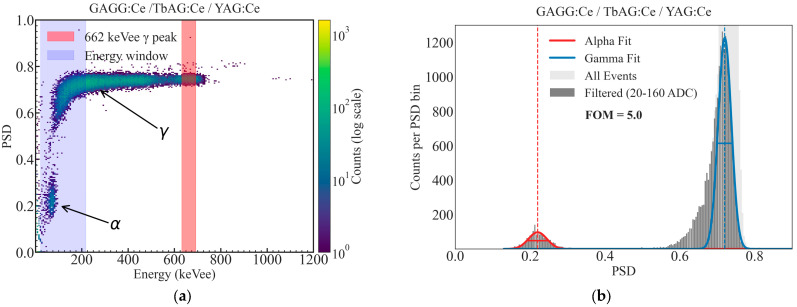
Two-dimensional PSD versus energy histogram (**a**) and corresponding one-dimensional PSD projection (**b**) with Gaussian fits and figure-of-merit (FOM) evaluation for YAG:Ce SCF/TbAG:Ce SCF/GAGG:Ce SC double-film composite scintillator measured using ^241^Am and ^137^Cs sources.

Using the optimized integration gates, two-dimensional PSD density plots were constructed as a function of the calibrated energy and PSD parameter, following the PSD parameter definition (Equation (1), Section 2.3.3). Representative PSD maps for the GAGG:Ce SC substrate, the TbAG:Ce SCF/GAGG:Ce SC single-film composite scintillator, and the YAG:Ce SCF/TbAG:Ce SCF/GAGG:Ce SC double-film composite scintillator are shown in Figure 14, Figure 15 and Figure 16. In all configurations, distinct α- and γ-events form clearly separated bands in the PSD space.

The bare substrate exhibits a reference level of α–γ separation. The addition of a TbAG:Ce SCF component enhances this separation, while the double-film composite structure with TbAG:Ce and YAG:Ce SCFs produces the most pronounced discrimination, yielding well-defined and widely separated α- and γ-induced event clusters. The 662 keV full-energy peak of ^137^Cs is clearly identifiable in the PSD maps and serves as a reference for comparison across samples. The observed improvement in PSD performance with increasing structural complexity is attributed to the modified scintillation decay-time components and enhanced temporal differentiation introduced by the SCF composite architecture.

## 4. Discussion and Conclusions

In this work, advanced composite scintillators based on single-crystalline films (SCFs) of TbAG:Ce and YAG:Ce grown on bulk GAGG:Ce single-crystal substrates were successfully designed, fabricated, and comprehensively characterized for enhanced α–γ discrimination in mixed radiation fields. The SCFs were deposited by the liquid-phase epitaxy (LPE) method, ensuring high crystalline quality, sharp epitaxial interfaces, a controlled thickness (10 µm TbAG:Ce and 20 µm YAG:Ce), and excellent optical coupling with the 500 µm Czochralski-grown GAGG:Ce substrate. Two architectures were realized: a TbAG:Ce SCF/GAGG:Ce SC single-film composite scintillator and a YAG:Ce SCF/TbAG:Ce SCF/GAGG:Ce SC double-film composite scintillator. The design was intentionally engineered to optimize energy deposition and introduce controlled differences in scintillation decay kinetics between low-penetrating α-particles and highly penetrating γ-rays.

Optical absorption, cathodoluminescence (CL), and X-ray-excited radioluminescence (RL) measurements confirmed that all structures exhibited dominant Ce^3+^ 5d_1_ → 4f (^2^F_5_/_2_, ^2^F_7_/_2_) emission characteristic of their respective garnet hosts. The introduction of TbAG:Ce and YAG:Ce SCFs modified both the absorption features and emission peak positions due to differences in crystal field strength and Stokes shifts among the garnet compositions. Under RL excitation, the luminescence response remains largely governed by the GAGG:Ce substrate, while the SCF components contribute measurable spectral shifts and kinetic modifications.

Scintillation studies under 5.5 MeV α-particles (^241^Am) and 662 keV γ-rays (^137^Cs) revealed the strong dependence of the light yield and decay kinetics on the composite architecture. Under α-excitation, the deposited SCFs strongly influence the response due to the complete absorption of α-particles within the thin films. The relative light yield decreases significantly from 100% for the GAGG:Ce substrate to 17% for the TbAG:Ce SCF/GAGG:Ce SC single-film composite scintillator and to 5.8% for the YAG:Ce SCF/TbAG:Ce SCF/GAGG:Ce SC double-film composite scintillator, demonstrating that α-energy deposition occurs predominantly within the SCF regions. In contrast, under γ-ray excitation, the response is largely governed by the GAGG:Ce substrate, yielding relative light yield values of 100% for the substrate, 75% for the TbAG:Ce SCF/GAGG:Ce SC single-film composite scintillator, and 73% for the YAG:Ce SCF/TbAG:Ce SCF/GAGG:Ce SC double-film composite scintillator. The reduced light yield in the SCFs is attributed to Pb^2+^-related quenching centers formed during LPE growth from PbO-based fluxes, which partially suppress Ce^3+^ emission.

Time-resolved scintillation measurements further highlight the impact of the composite design. While the γ-induced decay kinetics remain comparable across all samples, the α-induced decay profiles differ markedly between the GAGG:Ce substrate, the TbAG:Ce SCF/GAGG:Ce SC single-film composite scintillator, and the YAG:Ce SCF/TbAG:Ce SCF/GAGG:Ce SC double-film composite scintillator. The TbAG:Ce SCF/GAGG:Ce SC single-film composite scintillator exhibits a dominant slow component under α-excitation (mean decay time ~707 ns), whereas the YAG:Ce SCF/TbAG:Ce SCF/GAGG:Ce SC double-film composite scintillator shows a dominant fast component (mean decay time ~205 ns). This pronounced modification of the decay characteristics forms the physical basis for pulse-shape discrimination.

Energy calibration using ^57^Co, ^51^Cr, and ^137^Cs γ-ray sources enabled the conversion of detector signals to a calibrated keVee scale and the precise selection of energy windows for discrimination analysis. Systematic optimization of charge comparison integration gates demonstrated that the short gate width critically determines discrimination performance, while the long gate captures slower scintillation components. The figure of merit (FOM) for α–γ separation increases progressively with the structural complexity: 1.4 for the GAGG:Ce substrate, 1.9 for the TbAG:Ce SCF/GAGG:Ce SC single-film composite scintillator, and 5.0 for the YAG:Ce SCF/TbAG:Ce SCF/GAGG:Ce SC double-film composite scintillator. Two-dimensional PSD density maps clearly show well-separated α- and γ-populations, with the most distinct separation observed for the double-film structure.

For comparison, in our previous work [28], TbAG-based SCF/GAGG:Ce composite scintillators exhibited maximum FOM(PSD) values of 2.1(1), while lower values of 1.5(1) and 1.4(1) were obtained for other configurations and for the GAGG:Ce substrate. The present results for the substrate and single-film composite are consistent with these earlier findings, whereas the double-film composite structure demonstrates a substantially improved FOM of 5.0. This significant enhancement highlights the advantage of the multilayer design, in which the additional YAG:Ce film introduces a stronger temporal contrast between α- and γ-induced scintillation signals, leading to superior pulse-shape discrimination performance compared to previously reported garnet-based composite scintillators.

Overall, the results demonstrate that the epitaxial engineering of garnet-based composite scintillators provides a powerful pathway to tailoring scintillation decay kinetics, differential light yields, and time-response characteristics. The SCF-based composite architecture significantly enhances α–γ discrimination while maintaining efficient γ-ray detection, which is dominated by the bulk substrate. In particular, the YAG:Ce SCF/TbAG:Ce SCF/GAGG:Ce SC configuration achieves outstanding PSD performance, confirming the effectiveness of multilayer LPE-grown composites for mixed-field radiation detection.

Despite these promising results, several practical limitations should be considered. The reduced light yield of the SCF layers remains a key challenge due to Pb^2+^-related quenching centers introduced during LPE growth, which partially suppress Ce^3+^ emission, especially under α-excitation, where energy deposition is localized in the films. In addition, the reproducibility of SCF properties is sensitive to growth parameters such as temperature stability, the melt composition, and the growth rate, which may influence the film thickness, dopant distribution, and defect formation. The scalability of multilayer structures also remains technologically demanding, as precise control of sequential epitaxial growth steps is required for large-area or more complex architectures. A trade-off between the light yield and discrimination performance is therefore inherent in the current design strategy.

To address these aspects, further optimization of the LPE growth conditions, film composition, and activator concentration is required. The stability and reproducibility of the LPE process, together with its inherent scalability, further support the practical applicability of the developed composite scintillators. The consistent scintillation performance observed across independently prepared samples confirms the reliability of the fabrication method and highlights its suitability for producing large-area and multilayer detector architectures.

Overall, this study establishes LPE-fabricated garnet composite scintillators as highly promising candidates for applications requiring reliable particle identification, including nuclear spectroscopy, radiation monitoring, homeland security, and dosimetry in complex radiation environments.

## Figures and Tables

**Table 1:** Parameters of the composite scintillators. * Relative LYs were measured with 12 μs shaping time constant in comparison with standard YAG:Ce SCF with LY of 2650 photons/MeV under α-particle excitation by ^239^Pu (5.15 MeV) source.

Scintillator (Label)	Thickness (µm)	Density ρ (g/cm^3^)	Z_eff_	ρ × Z_eff_ ^4^ (10^6^ g/cm^3^)	Melting Point (°C)	Ce Content (at.%)	Relative LY, % *
YAG:Ce SCF	20	4.56	26	2.08	970	0.11	95
TbAG:Ce SCF	10	6.07	34	13.4	995	0.098	175
GAGG:Ce SC (Substrate)	500	6.63	54.4	87.6	1850	0.018	325

^4^: Product of density (ρ) and effective atomic number (Zeff).

**Table 2:** Scintillation decay times and relative LYs of the GAGG:Ce SC substrate and the TbAG:Ce SCF/GAGG:Ce SC and YAG:Ce SCF/TbAG:Ce SCF/GAGG:Ce SC composite scintillators under α-particle excitation from a ^241^Am source (5.5 MeV) and γ-ray excitation from a ^137^Cs source (662 keV).

Sample	γ-Decay Components τ_γi_ (ns)	τ_γ_mean_ (ns)	α-Decay Components τ_αi_ (ns)	τ_α_mean_ (ns)	LY_γ_ (662 keV), %	LY_α_ (5.5 MeV), %
GAGG:Ce SC	253 (73%), 1030 (27%)	459	303 (83%), 1180 (17%)	456	100	100
TbAG:Ce SCF/ GAGG:Ce SC	309 (76%), 770 (24%)	420	205 (8%), 752 (92%)	708	75	17
YAG:Ce SCF/TbAG:Ce SCF/GAGG:Ce SC	255 (36%), 825 (64%)	460	50 (71%), 582 (29%)	205	73	5.8

## Data Availability

The original contributions presented in this study are included in the article. Further inquiries can be directed to the corresponding authors.

## References

[1] Ferrand B., Chambaz B., Couchaud M. (1999). Liquid phase epitaxy: A versatile technique for the development of miniature optical components in single crystal dielectric media. Opt. Mater..

[2] Nikl M. (2016). Nanocomposite, Ceramic, and Thin Film Scintillators.

[3] Robertson J.M., van Tool M.V. (1984). Cathodoluminescent garnet layers. Thin Solid Film..

[4] Nikl M., Yoshikawa A. (2015). Recent R&D trends in inorganic single-crystal scintillator materials for radiation detection. Adv. Opt. Mater..

[5] Molva E. (1999). Microchip lasers and their applications in optical microsystems. Opt. Mater..

[6] Klimczak M., Malinowski M., Sarnecki J., Piramidowicz R. (2009). Luminescence properties in the visible of Dy:YAG/YAG planar waveguides. J. Lumin..

[7] Koch A., Raven C., Spanne P., Snigirev A. (1998). X-ray imaging with submicrometer resolution employing transparent luminescent screens. J. Opt. Soc. Am. A.

[8] Martin T., Koch A. (2006). Recent developments in X-ray imaging with micrometer spatial resolution. J. Synchrotron Radiat..

[9] Derenzo S.E., Moses W.W., Cahoon J.L., Perera R.C.C., Litton J.E. (1990). Prospects for new inorganic scintillators. IEEE Trans. Nucl. Sci..

[10] Bachiri A., Abouais A., Trykowski G., Boumhamdi M., Kamiński D., Guerchi A., Strzałkowski K., Drozdowski W., Świderski Ł., Syntfeld-Każuch A. (2026). Influence of beryllium on thermal properties and lattice disorder in ZnSe bulk crystals. Thermochim. Acta.

[11] Abouais A., Boumhamdi M., Laouid A., Belghiti A.A., Strzałkowski K., Singh D., Trykowski G., Hajjaji A. (2026). Growth and thermal characterization of Zn_1−x_Mn_x_Se crystals. J. Therm. Anal. Calorim..

[12] Kamada K., Yanagida T., Pejchal J., Nikl M., Endo T., Tsutsumi K., Fujimoto Y., Fukabori A., Yoshikawa A. (2012). Crystal growth and scintillation properties of Ce-doped Gd_3_(Ga,Al)_5_O_12_ single crystals. IEEE Trans. Nucl. Sci..

[13] Fasoli M., Vedda A., Nikl M., Jiang C., Uberuaga B.P., Andersson D.A., McClellan K.J., Stanek C.R. (2011). Band-gap engineering for removing shallow traps in rare-earth Lu_3_Al_5_O_12_ garnet scintillators using Ga^3+^ doping. Phys. Rev. B.

[14] Bachiri A., Makowski M., Witkowski M.E., Drozdowski W., Gałazka Z. (2023). Assessment of the scintillation properties of MgGa_2_O_4_ and ZnGa_2_O_4_ single crystals. Opt. Mater. Express.

[15] Oya T., Nakauchi D., Okada G., Kawaguchi N., Yanagida T. (2017). Scintillation properties of Ce-doped Tb_3_Al_5_O_12_. Nucl. Instrum. Methods Phys. Res. A.

[16] Moszyński M. (2005). Energy resolution of scintillation detectors. Proc. SPIE.

[17] Dujardin C., Auffray E., Bourret-Courchesne E., Dorenbos P., Lecoq P., Nikl M., Vasil’ev A.N., Yoshikawa A., Zhu R.-Y. (2018). Needs, trends, and advances in inorganic scintillators. IEEE Trans. Nucl. Sci..

[18] Kamada K., Shoji Y., Kochurikhin V.V., Nagura A., Okumura S., Yamamoto S., Yeom J.Y., Kurosawa S., Pejchal J., Yokota Y. (2017). Single crystal growth of Ce:Gd_3_(Ga,Al)_5_O_12_ with various Mg concentration and their scintillation properties. J. Cryst. Growth.

[19] Lecoq P. (2016). Development of new scintillators for medical applications. Nucl. Instrum. Methods Phys. Res. A.

[20] Nargelas S., Skruodienė M., Solovjovas A., Ščefanavičius Š., Soltanaitė G., Migauskas M., Podlipskas Ž., Kareiva A., Tamulaitis G. (2025). Novel scintillators based on cerium-doped garnets in amorphous silica: Crystal quality at the cost of glass. J. Mater. Chem. C.

[21] Syntfeld-Każuch A., Szczęśniak T., Bachiri A., Brylew K., Gorbenko V.I., Zorenko T., Syrotych Y., Sidletskiy O., Zorenko Y. (2026). Characterization of Novel Composite Scintillators Based on the Epitaxial Structures of TbAG:Ce/GAGG:Ce and TbAG:Ce,Mg/GAGG:Ce Garnets in Mixed Radiation Fields. Crystals.

[22] Guzik Z., Borsuk S., Traczyk K., Płomiński M. (2006). TUKAN-an 8K pulse height analyzer and multi-channel scaler with a PCI or a USB interface. IEEE Trans. Nucl. Sci..

[23] CAEN (2019). User Manual and Specifications.

[24] Glass H.L., Elliott M.T. (1974). Accommodation of Pb in yttrium iron garnet films grown by liquid phase epitaxy. J. Cryst. Growth.

[25] Kummer F., Zwaschka F., Ellens A., Debray A., Waitl G. (2001). Luminous Substance for a Light Source and Light Source Associated Therewith. International Patent Application.

[26] Batentschuk M., Osvet A., Schierning G., Klier A., Schneider J., Winnacker A. (2004). Simultaneous excitation of Ce^3+^ and Eu^3+^ ions in Tb_3_Al_5_O_12_. Radiat. Meas..

[27] Zorenko Y., Gorbenko V., Voznyak T., Zorenko T. (2008). Luminescence of Pb^2+^ ions in YAG:Pb single-crystalline films. Phys. Status Solidi B.

[28] Zatryb G., Klak M.M. (2020). On the choice of proper average lifetime formula for an ensemble of emitters showing non-single exponential photoluminescence decay. J. Phys. Condens. Matter.

